# Supplementary material for: Maternal health and health-related behaviours and their associations with child health: Evidence from an Australian birth cohort
Source: PLoS One. 2021 Sep 13;16(9):e0257188. doi: 10.1371/journal.pone.0257188 (PMC8437270; doi:10.1371/journal.pone.0257188)
Supplement: Attachment — Submitted filename: Response to Reviewers - updated.docx [file pone.0257188.s001.docx]

**Response to the reviewer’s comments**

| Academic Editor | | | | |
| --- | --- | --- | --- | --- |
| No. | Editor’s comments | Author’s response | References |  |
| 1. | 1. Please ensure that your manuscript meets PLOS ONE's style requirements, including those for file naming. The PLOS ONE style templates can be found at  <https://journals.plos.org/plosone/s/file?id=wjVg/PLOSOne_formatting_sample_main_body.pdf> and  <https://journals.plos.org/plosone/s/file?id=ba62/PLOSOne_formatting_sample_title_authors_affiliations.pdf> | The corresponding author reviewed and revised the styles as per the requirement of PLOS ONE, which include reference style, file naming and other requirements as specified by the editor. | N/A |  |
| 2. | 2. We note that you have indicated that data from this study are available upon request. PLOS only allows data to be available upon request if there are legal or ethical restrictions on sharing data publicly. For more information on unacceptable data access restrictions, please see <http://journals.plos.org/plosone/s/data-availability#loc-unacceptable-data-access-restrictions>.   In your revised cover letter, please address the following prompts:  a) If there are ethical or legal restrictions on sharing a de-identified data set, please explain them in detail (e.g., data contain potentially sensitive information, data are owned by a third-party organization, etc.) and who has imposed them (e.g., an ethics committee). Please also provide contact information for a data access committee, ethics committee, or other institutional body to which data requests may be sent.  b) If there are no restrictions, please upload the minimal anonymized data set necessary to replicate your study findings as either Supporting Information files or to a stable, public repository and provide us with the relevant URLs, DOIs, or accession numbers. | In the revised cover letter, following data availability statement has been provided:  “Data used in this study can be accessed through contacting the [Longitudinal Study of Australian Children Dataverse](https://dataverse.ada.edu.au/dataverse/lsac)  of National Centre for Longitudinal Data, Australian Government Department of Social Services. One can also email to [ada@anu.edu.au](mailto:ada@anu.edu.au) requesting data access. The lead author was granted permission to the data through online application from the following web link: <https://growingupinaustralia.gov.au/data-and-documentation/accessing-lsac-data>. ” |  |  |
| 3. | 3. Thank you for stating the following in the Financial Disclosure section:  'This research did not receive any specific grant from funding agencies in public, commercial or not-for-profit sectors. The corresponding author (KA) has affiliation from commercial organization, Purple Informatics (PI). KA is a consultant of the commercial affiliation, PI. However, PI did not provide any fund for this paperwork, and did not have any role in the study design, data collection and analysis, decision to publish or preparation of the manuscript for this study. Rather, this study is a part of PhD study of the author, KA.'  We note that one or more of the authors are employed by a commercial company: Purple Informatics.   a. Please provide an amended Funding Statement declaring this commercial affiliation, as well as a statement regarding the Role of Funders in your study. If the funding organization did not play a role in the study design, data collection and analysis, decision to publish, or preparation of the manuscript and only provided financial support in the form of authors' salaries and/or research materials, please review your statements relating to the author contributions, and ensure you have specifically and accurately indicated the role(s) that these authors had in your study. You can update author roles in the Author Contributions section of the online submission form.  Please also include the following statement within your amended Funding Statement.  “The funder provided support in the form of salaries for authors [insert relevant initials], but did not have any additional role in the study design, data collection and analysis, decision to publish, or preparation of the manuscript. The specific roles of these authors are articulated in the ‘author contributions’ section.”  If your commercial affiliation did play a role in your study, please state and explain this role within your updated Funding Statement.   b. Please also provide an updated Competing Interests Statement declaring this commercial affiliation along with any other relevant declarations relating to employment, consultancy, patents, products in development, or marketed products, etc.  Within your Competing Interests Statement, please confirm that this commercial affiliation does not alter your adherence to all PLOS ONE policies on sharing data and materials by including the following statement: "This does not alter our adherence to  PLOS ONE policies on sharing data and materials.” (as detailed online in our guide for authors [http://journals.plos.org/plosone/s/competing-interests](about:blank)) . If this adherence statement is not accurate and  there are restrictions on sharing of data and/or materials, please state these. Please note that we cannot proceed with consideration of your article until this information has been declared.   c. Please include both an updated Funding Statement and Competing Interests Statement in your cover letter. We will change the online submission form on your behalf. | As suggested, the following amended Funding Statement has been mentioned in the cover letter and also at the online submission:  “Please note that, this research did not receive any specific grants from any funding agencies in public, commercial or not-for-profit sectors. The corresponding author (KA) has affiliation from commercial organization, Purple Informatics (PI). KA is a consultant of the commercial affiliation, PI. The funder provided support in the form of consultancy fees for the author “KA” for other works but did not provide any financial support for this work. Further, the funder (PI) did not have any additional role in the study design, data collection and analysis, decision to publish, or preparation of the manuscript. The specific roles of these authors are articulated in the ‘author contributions’ section.”  Also, provided the following updated competing interest declaration in the cover letter and online submission:  “This study is a part of PhD study of the author, KA. The corresponding author’s affiliation to PI does not alter the authors adherence to PLOS ONE policies on sharing data and materials. Other authors do not have any competing interests.” | Cove Letter |  |
| 4. | 4. Please include a copy of Tables 1, 2, 3 and 4 which you refer to in your text on pages 9, 10 and 11. | In the earlier submission, tables were not attached mistakenly. However, in the revised submission, the tables have been presented in the manuscript file as per the requirement of the PLOS ONE submission guideline. |  |  |
|  | Additional Editors Comments: |  |  |  |
| 5. | Additional Editor Comments:  The manuscript appears to be missing all tables and figure. Please include these in the submission to allow us to evaluate your manuscript.   [Note: HTML markup is below. Please do not edit.]  Reviewers' comments:  N/A | Thanks so much for locating the error. Mistakenly we did not attached the tables earlier. However, in this revised submission we have presented the tables in the manuscript file.  Please note that this study has no figure, hence no figure file was uploaded. |  |  |
