# Supplementary material for: Maternal health and health-related behaviours and their associations with child health: Evidence from an Australian birth cohort
Source: PLoS One. 2021 Sep 13;16(9):e0257188. doi: 10.1371/journal.pone.0257188 (PMC8437270; doi:10.1371/journal.pone.0257188)
Supplement: Attachment — Submitted filename: Comments.docx [file pone.0257188.s002.docx]

The study by Ahmad et al. estimated the associations of several maternal health conditions and health related behaviors in pregnancy and 0-15 months after childbirth and child general health status, presence of chronic health conditions, and physical health outcome indices at age 0-1 and age 12-13, using data from an Australian representative household survey named the Longitudinal Survey of Australian Children. Please see my major and minor comments below:

Major comments:

1. About the study population: the definition of the analytic sample is not clear. In Line 122-123, it seems the only inclusion criterion is that biological mothers had responded as caregivers in the interview. From Table 2-4, the authors reported different numbers for the associations between health behavior related variables and child outcomes, which made me feel that they had different analytic samples for each table. If so, please make the definition of the analytic sample clear, and provide the N for each analysis in the tables.

2. About the dependent variables: The N of stressful life events in the past 12 months and psychological distress in the past 4 weeks could have different clinical meanings if a woman was interviewed during pregnancy or right after delivery vs. 1 year after delivery. Moreover, all these measurements are very vague, and it is difficult to pinpoint the specific health conditions with these measurements, therefore, to inform interventions. These limitations should be mentioned in the discussion.

3. About the independent variables: Same comment as above about the imprecise health conditions.

4. About the statistical methods:

1) The same set of covariates were adjusted in models with different exposures and outcomes. Mental health, medical conditions, and health behaviors can have distinct characteristics from each other, and it may be worth some careful consideration of different covariates being involved, if the data are available.

2) Gestational age and birthweight may serve as mediators between maternal health and child health. Please re-consider the adjustments of these two variables.

3) Although the study had two measurements of child outcome, there was no longitudinal or repeated measurement analysis being conducted. If this is not the research interest, please change the rationale in Line 95-96 – this study did not address the trajectory of health outcomes.

4) Maternal physical health, mental health, and health related behaviors may not occur and/or act independently. They may share biological mechanisms with each other, confound or mediate each other’s associations with child outcomes. There are advanced methods estimating the effects of overall exposure mixture rather than single exposures, such as weighted quantile sum regression or Bayesian kernel machine regression. If this is not the research interests, please at least consider having some of the dependent variables as potential confounders for the other dependent variables.

5) I find Table 2-4 very confusing. It seems the associations between maternal health behavior variables with the three outcomes were repeatedly measured in three different samples. The association reported in Line 289 – 1.48 (95%CI: 1.07-2.04) was not found in these three tables. I wonder what the rationale is to repeat the analysis in this way. Why not have one set of analysis for these variables in their maximum sample?

6) The authors perform many tests in this study. Would multiple testing be a concern that needs additional attention?

5. About the discussion: Is there any biological plausibility or social models explaining that poorer maternal health is adversely associated with child health, e.g., any laboratory science in animals or humans, or any conceptual models from sociology? It seems all the results are consistent with previous studies, and the discussion did not state the unique contribution of this study to this field of science clearly.

Minor comments:

1. The author needs to provide a table of the baseline characteristics of the mothers and children, such as maternal age, home language, etc. This information commonly goes in Table 1.

2. Please check whether breastfeeding is defined as one of the health-related behaviors or a confounder. It is shown in Table 2-4, but also mentioned as a confounder in the footnote of Table 2.

3. When the author mentioned “no evidence was found of multicollinearity” (Line 225-226), does it refer to the collinearity among dependent variables, independent variables, or the associations? How did they test the multicollinearity?

4. Since this study does aim to do causal inference, please avoid using the terms that indicate causal relationships, for example, Line 101, “investigating the impacts of”, and Line 297, “is harmful to”.
