# Supplementary material for: Maternal health and health-related behaviours and their associations with child health: Evidence from an Australian birth cohort
Source: PLoS One. 2021 Sep 13;16(9):e0257188. doi: 10.1371/journal.pone.0257188 (PMC8437270; doi:10.1371/journal.pone.0257188)
Supplement: Attachment — Submitted filename: Response to Reviewers.docx [file pone.0257188.s003.docx]

**Response to the reviewer’s comments**

| Academic Editor | | | | |
| --- | --- | --- | --- | --- |
| No. | Editor’s comments | Author’s response | References |  |
| 1. | I apologize for the delay in reaching a decision regarding your manuscript. This is due to difficulties in getting external reviewers. We have now received comments from two reviewers. They have some major concerns about your manuscript. I therefore ask that you to carefully address these comments before we can reach a final decision about your manuscript. | Thanks to the Academic Editor and the reviewers for providing constructive feedback on our paper. We have addressed all the feedback and hope the quality of this paper has now been improved substantially. |  |  |

**Response to the reviewer’s comments**

| Reviewer #1 | | | |
| --- | --- | --- | --- |
| No. | Reviewer’s comments | Author’s response | References |
|  | Ahmad et al. conducted a research on a topic of interest entitled "Maternal health and health-related behaviours and their associations with child health: evidence from an Australian birth cohort." The study investigated the association between maternal general health status, mental health and chronic illness conditions, and health-related behaviours during pregnancy and in the first year postdelivery, and three health outcomes during infancy and adolescent periods. I commend the authors for their interesting work on the topic, however, I have the following concerns. | Thank you so much for the rigorous feedback. As per the feedback, re-analysis was accomplished, and adjustment and improvement of the manuscript were performed. |  |
| 1. | 1. LSAC survey recruited infants (aged 0-1) and kindergarten kids (aged 4-5) along with their families in the first Wave (Wave 1) survey conducted in 2004. As the authors indicated those infants included in the first survey became adolescents (aged 12-13) during survey in Wave 7, similarly, kindergarten kids in Wave 1 became adolescents (aged 12-13) in Wave 5 survey conducted in 2012. However, while the present study accessed data LSAC surveys, the authors did not include a cohort of adolescents involved in 2012. This could have been due to that the kindergarten kid’s cohort database might not have provided information on maternal health status during pregnancy and postpartum period to be applicable for their study. This warrants some discussion in the methods section. | Our research objective was to investigate the association of mothers’ health and health related variables during pregnancy or in the year of childbirth and their offspring’s health outcomes during infancy and adolescence. Therefore, we select only B-cohort which has all relevant variables for both infancy and adolescence. K-cohort does not have any information of mother’s health and health related variables during pregnancy against the children’s infant age and also does not have any infant’s health outcome data to make a proper comparison of infancy and adolescence. Moreover, though mothers of K cohort provided information on maternal health, those would be retrospective, four years earlier and might have more recall bias. Hence, we excluded the K cohort from the analysis.  In the text, I have mentioned this in the method section as below:  “Although children of kindergarten cohorts (aged 4-5) in Wave 1 became adolescents (aged 12-13) in Wave 5, infant data of this cohort is not available. Moreover, although mothers of kindergarten cohort provided information on maternal health, those would be retrospective, four years earlier and might have more recall bias. Hence, this study did not use the data of kindergarten cohort from LSAC survey.” | Page 6, line 122 to 127. |
| 2. | 2. Considering the research questions studied in the study, it would be important for the authors to add some discussion related to the power and sample size of the study. | Thanks for the feedback. To address this issue, we examined, as per Bujang et al. (2018), the study’s sample size and found that it is sufficient for having required statistical power. Further, as per Rosner, B (2010) and Levine et al (2001), we did the post hoc calculation of statistical power and found that each of the three models of infants and adolescents has over 95% statistical power for the significant estimates and for some estimates it was over 99%. Moreover, the confidence intervals also affirm the statistical power. We have added the following text in the method section to inform about the power and sample size of the study:  “This study has sufficient statistical power regarding the sample size required to derive the statistics that represent the parameters or to avoid type 1 error of inference. Our study utilized logistic regression models for Table 2 and 3 with 14 independent variables from the observational surveys which require a minimum sample size of 800 as per the recommend rules of thumb explained by Bujang et al. (2018), while our study had over 5000 observations for infants and over 3000 observations for adolescents. Table 4 used linear regression model and its sample size was also sufficient to retain the expected level of statistical power in the analyses.” | Page 11-12, line 252 to 260 |
| 3. | 3. It was difficult to comprehend whether all or some of the key exposure variables collected maternal health information at during pregnancy or during the postpartum period in the paper. For example, as the presence of psychological distress for mothers assessed in “the past four weeks” (page 9, line 193), it was unlikely that the measures evaluated maternal mental health conditions to go back to the pregnancy period. Furthermore, the “number of stressful life events” for mothers would have covered time points back to the antepartum period. However, it was confusing when authors presented their results in one hand saying, “Psychological distress in the year after childbirth … (line 275-276) and on the other hand saying “… psychological distress during pregnancy…” (line 308). Please clarify the variables measurement and descriptions in the paper. | Thank you for pointing this. We have revised the text clarifying the variables measurement as follows:  “(iii) psychological distress experienced in the past four weeks. To measure psychological distress, mothers were asked the following questions to answer though recalling the past four week from the time of interviews while their children were 3 to 15 months old.” (Page 9-10, line 201-204)  “Mothers having stress, anxiety or depression during pregnancy (OR: 1.21 95% CI: 0.97–1.50) and psychological distress in past 4 weeks from the interview in the year of childbirth (OR: 1.22, 95% CI: 1.05–1.42) were also associated with the presence of a chronic health condition among the infants. However, these risk factors did not have any significant associations for the adolescents (for example, OR 1.11, 95% CI: 0.84-1.48).” (Page 15, line 294-299) | Page 9-10, line 201-204 and  Page 15, line 294-299 |
| 4. | 4. Despite the authors adjusted for key maternal demographic and birth related confounding variables, they did not provide the distributions of these maternal and birth characteristics so that someone is be able to look the distributions of these confounders in the study population. | In line with your comments, we have revised Table 1. | Added in Table 1 |
| 5. | 5. While the current study provided brief information about the foetal origins of adult diseases that is used to explain some the potential associations between poor foetal health conditions and occurrence of chronic conditions later in life in the introduction section, their discussion was not sufficient to describe the potential mechanism/pathways to explain the associations revealed in their study. | As per the comment, we have improved the discussion part substantially. Please scrutiny the discussion part of the manuscript for reference. | Page 20-24, line 351-456 |
| 6. | 6. It is also worthwhile to set a stage to explain why some of the exposures were more important for outcomes during infancy but not during adolescent period and vice versa. | Thanks so much for raising the issue. As per the concern, result and discussion part of the manuscript has been improved with relevant text, some of which are as follows:  “The associations revealed in Table 2 were medium to large in effect size, while the magnitudes were higher in the association of poor maternal general health and poor general health of children for infants, compared to adolescents.” (Page 15, line 286-288)  “The associations revealed in Table 3 were small to medium in effect size, while the magnitude was higher for adolescents.” (Page 15, line 298-299)  “However, in general, our study reveals that the odds of having poor general health during infancy is greater while the odds of this health outcome is small for adolescents. Moreover, the current study reveals the associations between mother’s poor general health and children’s chronic illness and lower physical health index score, but this association is only during the adolescence period.” (Page 20-21, line 372-376)  “This study results reveal that stressful life events faced by mothers in 12 months prior interview in the year of childbirth or the stress, anxiety or depression during pregnancy increase the odds of child health in the long run (among adolescents for poor general health). On the contrary, anxiety or depression during pregnancy or psychological distress in the year of childbirth increase the odds of child health immediately (among infants for chronic illness or poor physical health index score), but in the long run it diminishes. This might relate to chronic illness during infancy which may imprint a long-term consequence of poor general health perception among the parents and children, although by adolescence those children were able to self-manage the chronic illness.” (Page 21, line 389-297) | Page 15, line 284-286  Page 15, line 298-299  Page 20-21, line 372-376  Page 21, line 389-297 |
| 7. | 7. It is also likely that adolescent health outcomes can be affected (confounded) by their health-related behaviours and environmental exposures during the adolescent period. However, these potential confounders and how these factors would influence the results presented in the study were not discussed. | Thank you. As per your comment and the concern of reviewer #2, we have added several health-related behavioural variables in the adolescent models. The variables have been shown in Table 1. Further, in the method and discussion sections relevant text has been included, shown as follows.  “It is expected that adolescent health outcomes could be confounded by their health-related behaviours. Hence, for the adolescent models, the following addition control variables were considered: physical activity, smoking, alcohol consumption, and how often they took fruits or vegetables in the previous day from the interview date. Distribution of all the control variables has been shown in Table 1.” (Page 11, line 237-241)  “Further, for adolescent health models this study adjusted for adolescent health behaviours related variables, which are available in LSAC, to make the estimates more reliable.” (Page 24, line 439-440) | Table 1  Page 11, line 237-241  Page 24, line 439-440 |

**Response to the reviewer’s comments**

| Reviewer #2 | | | |
| --- | --- | --- | --- |
| No. | Reviewer’s comments | Author’s response | References |
|  | Overall:  The study by Ahmad et al. estimated the associations of several maternal health conditions and health related behaviors in pregnancy and 0-15 months after childbirth and child general health status, presence of chronic health conditions, and physical health outcome indices at age 0-1 and age 12-13, using data from an Australian representative household survey named the Longitudinal Survey of Australian Children. Please see my major and minor comments below: | Thanks for the feedback. We express our gratitude to the reviewer for the time and effort. All the comments we received on this paper have been taken into account to improve the quality of the article, and we present our reply to each of them in the following. |  |
|  | **Major Comments** |  |  |
| 1. | 1. About the study population: the definition of the analytic sample is not clear. In Line 122-123, it seems the only inclusion criterion is that biological mothers had responded as caregivers in the interview. From Table 2-4, the authors reported different numbers for the associations between health behavior related variables and child outcomes, which made me feel that they had different analytic samples for each table. If so, please make the definition of the analytic sample clear, and provide the N for each analysis in the tables. | Thanks for the feedback. As per the concerns, the sample size (n) for each of the tables has been mentioned in the text. Further, a figure has been added and it was mentioned in methodology, as shown below:  “The birth cohort surveyed in Wave 1 in 2004 included 5107 infants, 3381 (66.2%) of whom were retained as adolescents for Wave 7 in 2016. Of these children, this study included the 5019 infants and 3327 adolescents after excluding primary caregivers who were not biological mothers of the children. Details of the exclusion and attrition of families and total sample are shown in Fig 1.” | Page 6, line 127 to 131  See Tables 1-4 |
| 2. | 2. About the dependent variables: The N of stressful life events in the past 12 months and psychological distress in the past 4 weeks could have different clinical meanings if a woman was interviewed during pregnancy or right after delivery vs. 1 year after delivery. Moreover, all these measurements are very vague, and it is difficult to pinpoint the specific health conditions with these measurements, therefore, to inform interventions. These limitations should be mentioned in the discussion. | As per the comment, text in the discussion was revised as follows:  “…our study considers three different measures of maternal mental health either during pregnancy or when their children were 3 to 15 months old, as LSAC measures these variables from these timepoints. As a result, this study has been able to evaluate the associations with confounding influence of all three types of maternal mental health. However, all these three different measurements may create difficulty to focus the associations of the health outcomes by specific timepoints.” | Page 22, line 404 to 409 |
| 3. | 3. About the independent variables: Same comment as above about the imprecise health conditions. | To address this concern, the following text has been added in the methodology section:  “It is worthy to mention that this study used the independent variables of maternal physical and mental health and health-related behaviours either during pregnancy or when their children were 3 to 15 months old, as LSAC measures these variables from these timepoints. Hence, the associations derived from these variables refer the odds of child health outcomes against the respective time points’ maternal health or health-related behaviours” | Page 10, line 218 to 222 |
| 4. | 4. About the statistical methods:  1) The same set of covariates were adjusted in models with different exposures and outcomes. Mental health, medical conditions, and health behaviors can have distinct characteristics from each other, and it may be worth some careful consideration of different covariates being involved, if the data are available. | As the health-related behaviours’ variables for adolescents are available, adolescent models have been adjusted with these additional covariates and the models have been re-analysed. | See Table 1-4. |
| 5. | 2) Gestational age and birthweight may serve as mediators between maternal health and child health. Please re-consider the adjustments of these two variables. | Thank you. These two variables have now been considered as explanatory variables and, in the methodology and discussion, the following text has been included:  “***Other Health Variables***  Further, this study utilised the variables birth weight status (<2500 gm = 1, >2500 gm = 0) and gestational age (<37 weeks = 1 , >=37 weeks = 0) as explanatory variables to analyse the health outcomes of the children. This is because these variables play important role in maternal and child health.” (Page 10, line 227-230)  “The present study did not find any significant associations of low birth weight or gestational age less than 37 weeks with poor general health status or any chronic health conditions for infants and adolescents, except for a lower physical health outcome index score among infants. This may relate to the explanation by Dauglas and Gear (1976) in another study that a national survey can contribute little to the understanding of low birthweight children due to the small number of low birth weight children who suffer from problems of morbidities[37]. Our population-based study might be interpreted to indicate that low birthweight children are relatively low in numbers in relation to all births and contribute little to the total burden of children with morbidities in Australia. However, hospital-based studies with special interest groups or a subgroup of the population may have been reported with higher odds of child health outcomes for low birth weights and gestational age at birth. These two risk factors may have mediating effects on child health outcomes that need further research.” (Page 21, line 377-388) | Page 10, line 224-227  Page 21, line 377 to 388 |
| 6. | 3) Although the study had two measurements of child outcome, there was no longitudinal or repeated measurement analysis being conducted. If this is not the research interest, please change the rationale in Line 95-96 – this study did not address the trajectory of health outcomes. | Thanks for pointing out the error. As per the suggestion, the text has been changes as follows:  “While many studies have linked sub-optimal foetal environments with adult disease, few have focused on children in infancy [24] or during adolescence [1, 21], and even fewer studies have addressed the health outcomes at these time points via a longitudinal survey.” | Page 5, line 92 to 94 |
| 7. | 4) Maternal physical health, mental health, and health related behaviors may not occur and/or act independently. They may share biological mechanisms with each other, confound or mediate each other’s associations with child outcomes. There are advanced methods estimating the effects of overall exposure mixture rather than single exposures, such as weighted quantile sum regression or Bayesian kernel machine regression. If this is not the research interests, please at least consider having some of the dependent variables as potential confounders for the other dependent variables. | As per the suggestions, the study has considered having the dependent variables of other models as potential confounders for analysing the regression model and thus adjusted the analytical results. | Table 2 – 4. |
| 8. | 5) I find Table 2-4 very confusing. It seems the associations between maternal health behavior variables with the three outcomes were repeatedly measured in three different samples. The association reported in Line 289 – 1.48 (95%CI: 1.07-2.04) was not found in these three tables. I wonder what the rationale is to repeat the analysis in this way. Why not have one set of analysis for these variables in their maximum sample? | Thanks for the feedback. As per the suggestions, Table 2-4 have been reanalysed considering the health behavior related variables for every outcome variable. The results section has been updated accordingly. | Table 2-4  Results section:  Page 12-19, line 261 to 350 |
| 9. | 6) The authors perform many tests in this study. Would multiple testing be a concern that needs additional attention? | To address this concern, firstly we reduced the no. of comparisons by revising the analyses of Table 2 to 4. Hence multiple testing error has been reduced drastically. Moreover, we reported the p-value and effect size and discussed the scientific plausibility and supporting data from other studies to validate our study results. In line with these considerations, authors have added the following text in the discussion part.  “Multiple testing is another concern for this study as it tested the study sample three times for testing the three hypotheses regarding the child health outcomes for each of the infant and adolescent sample. To address this concern, we reported the significance level of p-values and discussed the effect size where it was possible to understand the statistical power of the analysis and also discussed the scientific plausibility and supporting data from other studies to validate our study results.” | Page 23-24, line 448 to 453 |
| 10. | 5. About the discussion: Is there any biological plausibility or social models explaining that poorer maternal health is adversely associated with child health, e.g., any laboratory science in animals or humans, or any conceptual models from sociology? It seems all the results are consistent with previous studies, and the discussion did not state the unique contribution of this study to this field of science clearly. | To address this concern, the authors have been revised the discussion part extensively to depict the contribution of this study more clearly. | See discussion part as a whole.  Page 20-24, line 351 to 456 |
|  | **Minor Comments** |  |  |
| 11. | 1. The author needs to provide a table of the baseline characteristics of the mothers and children, such as maternal age, home language, etc. This information commonly goes in Table 1. | As per the recommendation, this information has been provided in Table 1. | See Table 1 |
| 12. | 2. Please check whether breastfeeding is defined as one of the health-related behaviors or a confounder. It is shown in Table 2-4, but also mentioned as a confounder in the footnote of Table 2. | The study has considered the variable breastfeeding as confounder and hence Table 2-4 has been updated accordingly. | See Table 2-4 |
| 13. | 3. When the author mentioned “no evidence was found of multicollinearity” (Line 225-226), does it refer to the collinearity among dependent variables, independent variables, or the associations? How did they test the multicollinearity? | Thanks for pointing the requirement of further clarification. We did several collinearity diagnostic measures for the independent variables specified in each of the regression models. To clarify the tests, the following revised text has been added in the manuscript.  “This study also conducted several collinearity diagnostic measures, including variance inflation factors, tolerance and eigenvalues for the independent variables specified in each of the regression models; no evidence of multi-collinearity was found.” | Page 11, line 247 to 250 |
| 14. | 4. Since this study does not aim to do causal inference, please avoid using the terms that indicate causal relationships, for example, Line 101, “investigating the impacts of”, and Line 297, “is harmful to”. | Thank you for locating the errors.  The text, “investigating the impacts of”, has been replaced with “investigating the associations of”. (Page 5, line 99)  The text: “is harmful to” has been replaced with “increase the odds of poor health”. (Page 20, line 358) |  |
